# Correction: Using of human capital management in small and medium-sized enterprises in context of Industry 4.0

**DOI:** 10.1371/journal.pone.0335873

**Published:** 2025-10-31

**Authors:** The PLOS One Staff

## Notice of republication

This article was republished on October 17^th^, 2025, to correct an error in the article title. The publisher apologizes for the errors. Please download this article again to view the correct version. The originally published, uncorrected article and the republished, corrected articles are provided here for reference.

## Supporting information

S1 File Originally published, uncorrected article. (PDF)

S2 File Republished, corrected article. (PDF)
