# Supplementary material for: Using of human capital management in small and medium-sized enterprises in context of Industry 4.0
Source: PLoS One. 2025 May 2;20(5):e0320568. doi: 10.1371/journal.pone.0320568 (PMC12047808; doi:10.1371/journal.pone.0320568)
Supplement: Attachment — Submitted filename: Round1_Respond to Editor.docx [file pone.0320568.s001.docx]

**Editor**

When submitting your revision, we need you to address these additional requirements.

1.  Please ensure that your manuscript meets PLOS ONE's style requirements, including those for file naming. The PLOS ONE style templates can be found at

[https://journals.plos.org/plosone/s/file?id=wjVg/PLOSOne_formatting_sample_main_body.pdf](https://track.editorialmanager.com/CL0/https:%2F%2Fjournals.plos.org%2Fplosone%2Fs%2Ffile%3Fid=wjVg%2FPLOSOne_formatting_sample_main_body.pdf/1/010f019489c60b0f-1113968c-eae8-44b9-bef9-10a256cc0a40-000000/_-IaTuGxHB_OG4FmK07K9srkx9luGHha7JDaGIcc5y8=195)  and

[https://journals.plos.org/plosone/s/file?id=ba62/PLOSOne_formatting_sample_title_authors_affiliations.pdf](https://track.editorialmanager.com/CL0/https:%2F%2Fjournals.plos.org%2Fplosone%2Fs%2Ffile%3Fid=ba62%2FPLOSOne_formatting_sample_title_authors_affiliations.pdf/1/010f019489c60b0f-1113968c-eae8-44b9-bef9-10a256cc0a40-000000/hvZx_4csGO7S9xwcta_dUgbarWW5InExbNrHwItNTPY=195)

2. You indicated that ethical approval was not necessary for your study. We understand that the framework for ethical oversight requirements for studies of this type may differ depending on the setting and we would appreciate some further clarification regarding your research. Could you please provide further details on why your study is exempt from the need for approval and confirmation from your institutional review board or research ethics committee (e.g., in the form of a letter or email correspondence) that ethics review was not necessary for this study? Please include a copy of the correspondence as an ""Other"" file.

3. Thank you for stating the following financial disclosure:

“This article was supported by project Scientific Grant Agency of the Ministry of Education of the Slovak Republic VEGA 1/0273/22, by project Slovak Research and Development Agency APVV-20-0004 The Effect of an Increase in the Anthropometric Measurement of the Slovak Population on the Functional Properties of Furniture and the Business Processes.”

Please state what role the funders took in the study.  If the funders had no role, please state: "The funders had no role in study design, data collection and analysis, decision to publish, or preparation of the manuscript."

If this statement is not correct you must amend it as needed.

Please include this amended Role of Funder statement in your cover letter; we will change the online submission form on your behalf.

4. We note that you have indicated that there are restrictions to data sharing for this study. PLOS only allows data to be available upon request if there are legal or ethical restrictions on sharing data publicly. For more information on unacceptable data access restrictions, please see http://journals.plos.org/plosone/s/data-availability#loc-unacceptable-data-access-restrictions.

Before we proceed with your manuscript, please address the following prompts:

a) If there are ethical or legal restrictions on sharing a de-identified data set, please explain them in detail (e.g., data contain potentially identifying or sensitive patient information, data are owned by a third-party organization, etc.) and who has imposed them (e.g., a Research Ethics Committee or Institutional Review Board, etc.). Please also provide contact information for a data access committee, ethics committee, or other institutional body to which data requests may be sent.

b) If there are no restrictions, please upload the minimal anonymized data set necessary to replicate your study findings to a stable, public repository and provide us with the relevant URLs, DOIs, or accession numbers. For a list of recommended repositories, please see

https://journals.plos.org/plosone/s/recommended-repositories. You also have the option of uploading the data as Supporting Information files, but we would recommend depositing data directly to a data repository if possible.

We will update your Data Availability statement on your behalf to reflect the information you provide.

5. Please remove your figures from within your manuscript file, leaving only the individual TIFF/EPS image files, uploaded separately. These will be automatically included in the reviewers’ PDF.

6. Please review your reference list to ensure that it is complete and correct. If you have cited papers that have been retracted, please include the rationale for doing so in the manuscript text, or remove these references and replace them with relevant current references. Any changes to the reference list should be mentioned in the rebuttal letter that accompanies your revised manuscript. If you need to cite a retracted article, indicate the article’s retracted status in the References list and also include a citation and full reference for the retraction notice.

_________________________________________________________________________________

Dear Editor,

thank you for providing your valuable advice on the formal editing of our manuscript to meet the requirements of your journal Plos One. We have done everything to improve the quality of our manuscript and make it suitable for publication by you.

1. We have carefully reviewed your instructions for formatting and editing our article. We have followed the instructions we found on the link you provided.
2. We have supplemented the ethical statement with the relevant guidelines and laws that govern our research and that demonstrate that an Ethics Committee meeting is not necessary. The Ethics Committee of our institution (University of Žilina, Slovakia) does not provide any official statements.
3. We had to change the statement on funding for the manuscript (donors) because the projects that were listed ended on December 31, 2024. We have added new donors and the formula: “The donors had no role in the design of the study, collection and analysis of data, decision to publish or preparation of the manuscript.” We have also added this to the cover letter.
4. The data we collected from the respondents were collected only for the purpose of processing at the University of Žilina, as stated in the instructions that the respondents read. For this reason, we have stated that the data is only available upon request from one of the author members. We upload them to the system in the form of an attached Excel file as "Other".
5. We have removed the images from the manuscript and uploaded them only as a separate files in format .tif.

We carefully checked the references and checked all sources (websites, ISSN, ISBN, DOI) that we used. We added the necessary information to the references that had incomplete citations. We added 2 sources (no. 20 and no. 45) because the two sources that we had originally listed in the text of the manuscript (Siddiqui, 2012 and Moritz, 2003) did not have all the information correctly listed and it was no longer possible to find them after a time gap.

We have highlighted in blue in the manuscript all the changes made based on your recommendations or comments from the reviewers.

Have a beautiful day!

Best wishes,

Authors
